# Ingested hyaluronan moisturizes dry skin

**DOI:** 10.1186/1475-2891-13-70

**Published:** 2014-07-11

**Authors:** Chinatsu Kawada, Takushi Yoshida, Hideto Yoshida, Ryosuke Matsuoka, Wakako Sakamoto, Wataru Odanaka, Toshihide Sato, Takeshi Yamasaki, Tomoyuki Kanemitsu, Yasunobu Masuda, Osamu Urushibata

**Affiliations:** 1 R&D Division, Kewpie Corporation, 2-5-7, Sengawa-cho, Chofu-shi, Tokyo, Japan; 2 Department of Dermatology, Toho University Ohashi Medical Center, 2-17-6 Ohashi, Meguro-ku, Tokyo, Japan

**Keywords:** Hyaluronan, Hyaluronic acid, Ingestion, Dry skin, Moisturizing

## Abstract

Hyaluronan (HA) is present in many tissues of the body and is essential to maintain moistness in the skin tissues, which contain approximately half the body’s HA mass. Due to its viscosity and moisturizing effect, HA is widely distributed as a medicine, cosmetic, food, and, recently marketed in Japan as a popular dietary supplement to promote skin moisture. In a randomized, double-blind, placebo-controlled clinical study it was found that ingested HA increased skin moisture and improved treatment outcomes for patients with dry skin. HA is also reported to be absorbed by the body distributed, in part, to the skin. Ingested HA contributes to the increased synthesis of HA and promotes cell proliferation in fibroblasts. These effects show that ingestion of HA moisturizes the skin and is expected to improve the quality of life for people who suffer from dry skin. This review examines the moisturizing effects of dry skin by ingested HA and summarizes the series of mechanisms from absorption to pharmacological action.

## Introduction

In 1934, Meyer *et al*. [1] isolated and identified hyaluronan (HA), also called hyaluronic acid, from the vitreous humor of a cow’s eyes. HA is a macromolecular mucopolysaccharide that is widely distributed in body tissues and intracellular fluids, and it is present at high concentrations in the synovial fluid, vitreous humor, and skin [2]. Because HA is highly viscous and retains moisture, it is responsible for facilitating smooth movement of the knee joint, and maintains the normal shape of the vitreous humor and moisturizes the skin. However, aging and extrinsic stimuli such as solar ultraviolet radiation, smoking, and air pollutants gradually reduces the amount of HA in the body [3-5]. The reduction of HA in the body increase joint pain and dry skin; however, it decreases skin tension. In recent years, the public has started to ingest HA to compensate for its loss in the body. Ingested HA reduces joint pain in the knees of patients with knee osteoarthritis [6,7]. Furthermore, HA dietary supplements are expected to be effective anti-aging supplements because an American ABC News program, which aired in November 2002, stated that the key to longevity in a specific Japanese village was their HA-rich diet [8].

Dry skin is not only caused by the hereditary factors but also by reduction of age-related decrease in intracellular lipid and naturally moisturizing factors such as free amino acids and specific salts in the stratum corneum [9,10]. In addition, the disturbance of the skin barrier by extrinsic stimuli such as a sudden change in the weather [11,12] and contact with chemical agents also induces dry skin [13,14]. Estimating the number of dry skin patients in Japan is difficult because several individuals with dry skin only visit the hospital when their symptoms are severe. However, several products and supplements for dry skin are sold in Japan, suggesting that many Japanese people seek treatment for their dry skin. Individuals with dry skin experience itching because the sensory nerves in the lower layer of the skin are directly subjected to the external stimuli by the collapsing skin barrier function. Dry skin decreases a person’s quality of life because of the discomfort associated with tight and dry skin. Topical moisturizers treat dry skin; however, the elderly and individuals living alone need assistance for applying the moisturizers because they cannot reach their backs. Thus, the improvement of dry skin by ingested HA is considered meaningful for those people.

Several Japanese studies have reported that ingested HA moisturizes dry skin [15-20]; however, these effects are rarely studied outside of Japan because only local researchers have access to this information. The use of HA as a dietary supplement is also relatively new compared with that of other nutrients used to treat dry skin; thus, the reports that examined the effects of ingested HA on the skin were compiled in this review to promote those studies. These studies elucidate the effects of ingested HA. Furthermore, HA can be utilized as a food constituent in the treatment of patients with dry skin.

This review discusses the efficacy of ingested HA in treating dry skin and identifies its mechanism of action.

### Hyaluronan

**Figure 1:**
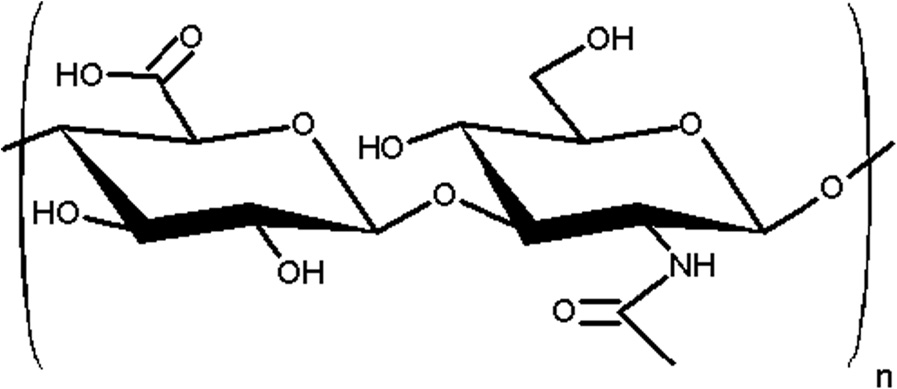
The structure of hyaluronan.

HA, a linear glycosaminoglycan is a major component of the extracellular matrix that is composed of repeating polymeric disaccharides of D-glucuronic acid and N-acetyl-D-glucosamine linked via alternating β-1, 4 and β-1, 3 glycosidic bonds [21] (Figure 1). HA exists in all vertebrates and in parts of microorganisms. In particular, more than 50% of total body HA is present in the skin of all vertebrates [22,23]. HA in the skin is synthesized by hyaluronan synthases (HAS) in epidermal keratinocytes and dermal fibroblasts [24,25]. The HA content of the dermis is far greater than that of the epidermis [26]; however, its function in the epidermis has not been elucidated. Previous studies reported that HA is closely involved in keratinocyte proliferation and differentiation [27-29] and may participate in epidermal structure and turnover. In the dermis, HA is responsible for regulating water balance and maintaining the cell structure by utilizing its high water retention and viscosity [30]. These facts suggest that HA is an important substance that maintains a healthy skin.

### Applications of hyaluronan

HA is typically used in medication, cosmetics, and food. Industrial applications use different molecular weights (MWs) of HA, which are divided into two broad categories on the basis of the method of HA preparation. These 2 categories are the chicken comb-derived HA and the HA derived from microbial fermentation. In recent years, increased mass production and the inexpensive cost of manufacturing has shifted HA production toward microbial fermentation.

Because the physical properties and physiological activities of HA differ depending on its MW [31], different MW products of HA, ranging from thousands to millions, are produced. In the field of medicine, high MW HA (approximately millions) is utilized to treat degenerative joint conditions because it has high viscosity and water retention; furthermore, it is used as an adjuvant treatment in cataract surgery [32-34]. Recently, as a treatment for facial lines and wrinkles, HA fillers in cosmetic surgery were used in the world. In the field of cosmetics, the proper MW of HA was selected from a wide range of MW (from hundreds of thousands to millions), which was determined by the type of cosmetic product. HA was formulated in a toner or an emulsion lotion depending on the intended use of the product. HA had a MW of millions or less, when it was formulated with common food such as bread and salad dressing.

In 1960, the first medication using HA was formulated; in 1979, the first cosmetic product using HA was manufactured. HA was sold as a food in 1942; thus, HA was used as a food long before it was used in medication or cosmetics. Endre Balazs applied for a patent in 1942 to commercially use HA as a substitute for egg whites in bakery products [35]. Since then, HA has been included in a variety of processed foods because of its physical and water retention properties [36,37].

People in Western Europe and China considered chicken combs, which contain a lot of HA, as an imperial cuisine. Further, Yang Guifei in China and Princess Catherine, the wife of King Henry II in medieval France, believed that if they ate chicken combs they would become beautiful. Even now, HA is ingested as a dietary supplement because it is expected to improve the skin and knee joints [8]. HA is approved as a food additive in Japan and Korea and as a health food in Korea. The United States of America, Canada, Italy and Belgium sell HA as a dietary supplement; however, in the United States, individuals consume HA to improve the health of their knee joints but the effects of HA on the skin are not well-known. The Japanese know that consuming HA improves skin health and knee joint pain; thus, the Japanese population spends approximately 250 million USD per year in 2012. These facts reveal that HA is widely used as a dietary supplement in Japan [38].

### Ingesting hyaluronan moisturizes the skin

Several studies have shown that ingested HA effects the knee joints [6,7] and the skin [15-20] (Table 1). In a randomized, double-blind, placebo-controlled study, human subjects that had chronically rough and dry skin (the average age ± S.E.; 26.7 ± 6.6) received 240 mg/day of HA (Hyaluronsan HA-F; MW 8 × 105, Kewpie Co., Tokyon = 11) for 6 weeks. Four evaluation phases by a dermatologist determined that the conditions of dry skin in face and whole body were significantly improved in the HA group respectively compared with the placebo group after 3 weeks and 6 weeks of ingestion. In addition, skin moisture content at the lower left part of the eye significantly improved in the HA group from 3 weeks to 6 weeks of ingestion, and skin smoothness in left of the upper arm and back of neck was significantly improved after 3 and 6 weeks of ingestion prior to the study [15]. Increases in skin moisture and improvements in skin condition in the placebo group were not confirmed compared with prior to its ingestion [15]. Further, 240 and 120 mg/day of HA ingestion revealed that these 2 doses had equivalent effects on the skin. Namely, a daily HA dose of 120 mg (n = 17) significantly increased skin moisture in the lower left part of the eye compared with a daily placebo dose (n = 18) 2 weeks after HA was consumed in a randomized, double-blind, placebo-controlled study for subjects with dry skin (average age ± S.E; 31.5 ± 13.3) [16].

**Table 1:** Summary on the skin improving effects of ingested hyaluronan

**Test method**	**Test design**	**Substance**	**Subjects**	**Results**	**References**
Oral consumption of HA at 240 mg daily for 6 weeks	Randomized, double-blind, placebo-controlled trial	HA (M.W.: 80 K)	22 patients with dry skin (in Japan)	Improved dry skin on the face and whole body	Kajimoto, O. *et al*. (2001) [15]
				Significant increase of skin moisture	
Oral consumption of HA at 120 mg daily for 4 weeks	Randomized, double-blind, placebo-controlled trial	HA (M.W.: 80 K)	35 patients with dry skin (in Japan)	Significant increase of skin moisture	Sato, T. *et al*. (2002) [16]
Oral consumption of HA at 120 mg daily for 6 weeks	Randomized, double-blind, placebo-controlled trial	HA (M.W.: 80 K)	39 female patients with dry skin (in Japan)	Significant increase of skin moisture	Sato, T. *et al*. (2007) [17]
Oral consumption of HA at 120 mg daily for 6 weeks	Randomized, double-blind, placebo-controlled trial	HA (M.W.: 30 K)	42 female patients with dry skin (in Japan)	Significant increase of skin moisture	Yoshida, T. *et al*. (2009) [18]
Oral consumption of HA at 37.52 mg daily for 30 days	Randomized, single-blind, placebo-controlled trial	Mixture containing HA (M.W. of HA: 2,500)	107 healthy subjects (in China)	Significant increase in skin moisture	Terashita, T. *et al*. (2011) [19]
				Significant increase in skin pH	
Oral consumption of HA at 100 mg daily for 12 weeks	Prospective open-label trial	Mixture containing HA(M.W.: unknown)	26 healthy female subjects (Caucasian, African-American, Hispanic, and others)	Improved aging symptoms on the face	Schwartz, S. R. *et al*. (2012) [20]

**Figure 2:**
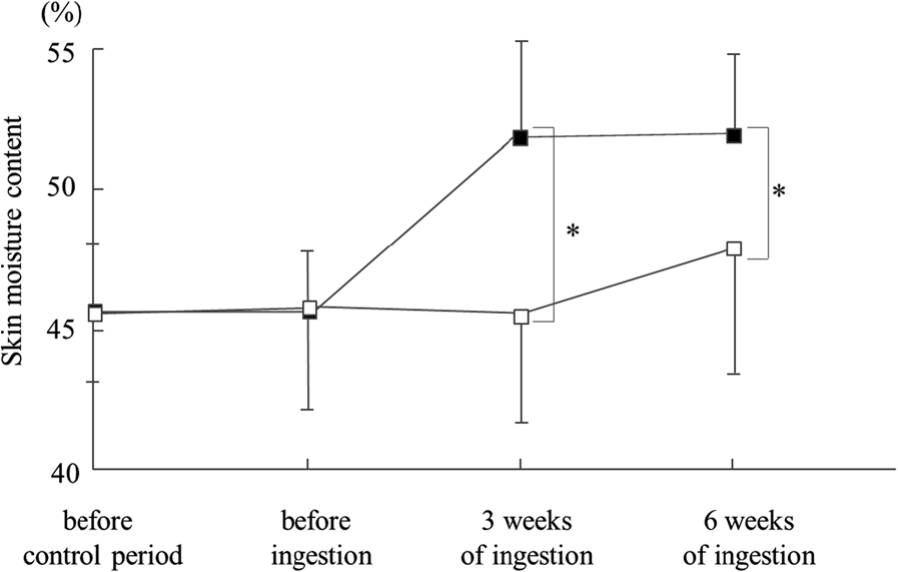
**Skin moisture content changes by HA (MW: 8 × 10**^**5**^**) ingestion or placebo.** 39 female subjects were randomly divided into two groups (HA group, n = 19; placebo group, n = 20) to minimize the inter-group differences in skin moisture content, skin elasticity and age. The skin moisture content at the lower part of the left eye was measured using a Corneometer® CM 825 before the control period, before the ingestion, and after 3 and 6 weeks of the ingestion. ■ indicates HA; □ indicates placebo. An unpaired *t*-test was used to compare the two groups. Data are presented as mean ± S.E. Asterisks indicate a statistically significant difference, *p < 0.05.

Because aging is associated with a decrease of HA in the skin [3-5], a randomized, double-blind, placebo-controlled study of middle-aged and elderly female subjects with dry skin (average age ± S.E.; 43.6 ± 4.6) was conducted [17]. The HA group (n = 19), which ingested 120 mg/day of HA, was found to have a significant increase of skin moisture, and a tendency for the skin moisture to increase in the face, compared with the placebo group (n = 20) after 3 and 6 weeks of ingestion (Figure 2) [17]. These reports show that consuming either 120 or 240 mg/day of HA contributes to increased skin moisture and improves dry skin. Thus, the lowest recommended dose of HA ingestion is 120 mg per day.

**Figure 3:**
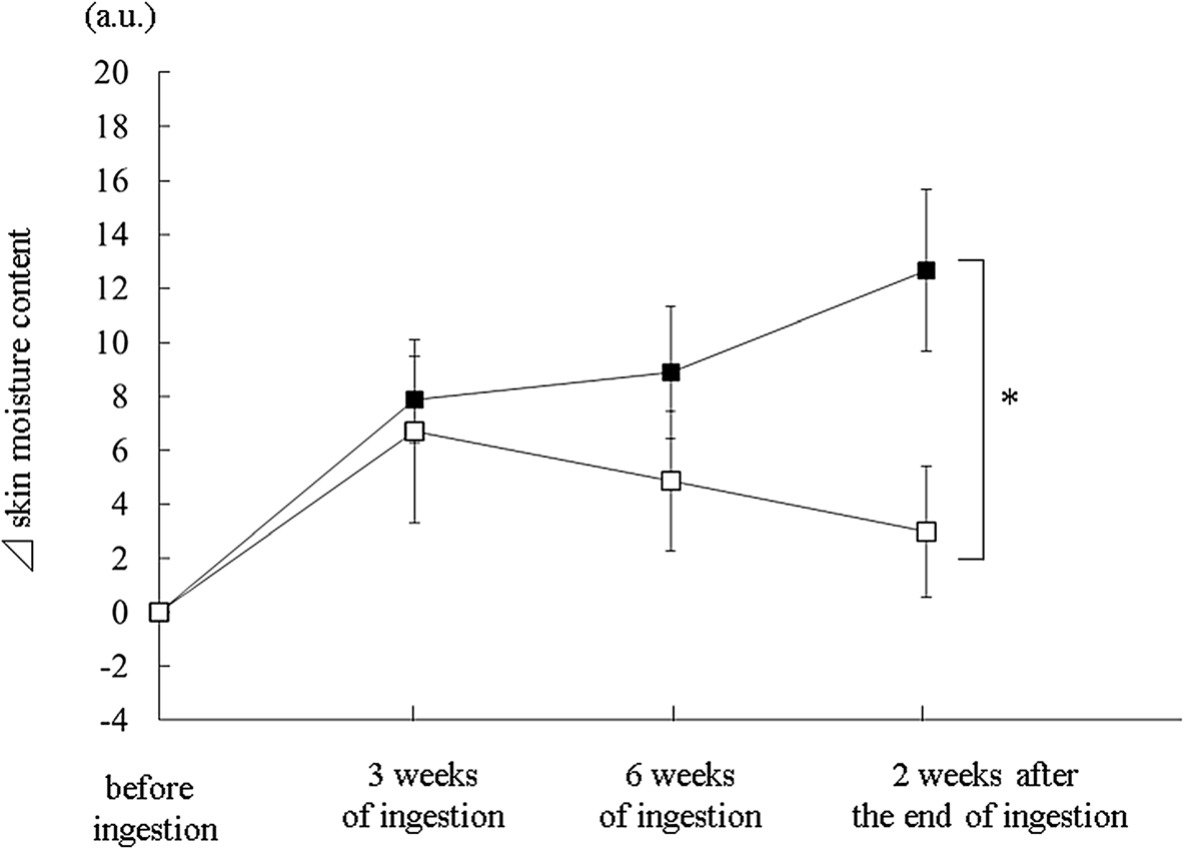
**Skin moisture content is changed by HA (MW: 3 × 10**^**5**^**) ingestion or placebo.** 42 female subjects were randomly divided into two groups (HA group, n = 20; subjects placebo group, n = 22) to minimize the inter-group differences in skin moisture content and age. The skin moisture content at the cheeks were measured with a Corneometer® CM 825 before the ingestion, after 3 and 6 weeks of the ingestion, and 2 weeks after the end of ingestion. Variations in the skin moisture content relative to baseline level are shown. ■ indicates HA; □ indicates placebo. An unpaired *t*-test was used to compare the groups. Data are presented as mean ± S.E. Asterisks indicate a statistically significant difference, *p < 0.05.

The aforementioned studies show that consuming HA derived from chicken combs moisturizes the skin. A report has revealed that consuming HA manufactured by fermentation also moisturizes the skin [18]. In a randomized, double-blind, placebo-controlled study, female subjects with dry skin (average age ± S.E; 43.3 ± 4.6) received 120 mg/day of HA (Hyabest®(S) LF-P: MW 3 × 10^5^, Kewpie Co., Tokyo, n = 20), or placebo (n = 22), for 6 weeks. The HA group had better skin moisture than the placebo group during the ingestion period. Furthermore, two weeks after HA ingestion, the HA group showed significant improvement in skin moisture compared with the placebo-controlled group (Figure 3) [18]. Because the skin’s turnover rate normally requires 28 days to complete, the effects of the ingested HA to the stratum corneum continued for 2 weeks after treatment. Thus, these results suggest that consuming HA moisturizes the skin for several weeks after the treatment has ended.

Dry skin is induced by a variety of factors. The reports summarized in this review reveal that consuming HA improves dry skin by increasing the skin’s moisture content. In addition, the skin-moisturizing effect of ingested HA is not dependent on the source or specific molecular size of the HA ingested.

There are morphological and characteristic differences in skin types among different races. In the skin of the white race, the stratum corneum is thicker and the amount of the sebum cutaneum is less than that of the black and the yellow races. The dermic layer in black and yellow races is thicker than that in the white race. The amount of sebum cutaneum increases with rising environmental temperatures [39] and this reveals that climate affects skin type. The aforementioned studies do not account for the different skin types or the environmental effects on the skin. Thus, there is some possibility that the skin-moisturizing effects of ingested HA is different according to the race of subjects or the climatic environment where they live.

In a study of non-Japanese subjects, dietary intake of chicken comb enzymatic decomposer containing HA (W-HA; mean M.W.: 2,500, Will Search Co. Ltd., Yokohama, Japan) 280 mg per day for 30 days significantly increased skin moisture and pH in a placebo-controlled single-blind clinical trial in the People’s Republic of China [19]. A pilot open-label study in 26 healthy females, including Caucasians and individuals of African and Hispanic descent, was carried out using chicken sternal articular cartilage hydrolyzed extract, BioCell Collagen® (BCC; BioCell Technology, LLC Newport Beach, CA), containing 100 mg of low-molecular-weight HA [20]. Daily supplementation with 1 g of BioCell Collagen® for 12 weeks led to a significant reduction of skin dryness and wrinkles, and a significant increase in the content of hemoglobin and collagen in the skin dermis. However, the quality of these trials is compromised by their prospective approach and test materials made of a crude extract; therefore, it would be insufficient to confirm the solitary effect of HA. Further study into racial variation by placebo-controlled double-blind clinical trials should be pursued to re-confirm the independent effect of HA.

Although there were limitations in the studies presented here, consuming HA moisturizes the skin. One of the factors that determines the smoothness and softness of bare skin [40] is the skin’s moisture content. Because consuming HA improves the skin’s moisture content, HA can improve the texture and reduce wrinkles in the skin. Although there were no significant differences between the skin texture and wrinkles in the dry skin of the HA group (Hyaluronsan HA-F, 120 mg/day) compared with the placebo group prior to the study, consuming HA significantly improved that 2 weeks after HA was ingested [15]. In addition, oral ingestion of HA (Hyabest®(S) LF-P) improved the skin’s moisture content in an ultraviolet irradiated skin model [41,42]. Features of skin aging, such as wrinkle formation, occur primarily because of irradiating ultraviolet rays from sunlight; thus, HA can also be expected to have anti-aging effects, such as improving skin texture and reducing wrinkles.

Furthermore, dry skin causes itching in patients with atopic dermatitis and senile xerosis. Thus, consuming HA moisturizes the skin and also reduces the itching that is associated with dry skin.

### Pharmacokinetics of ingested hyaluronan

**Figure 4:**
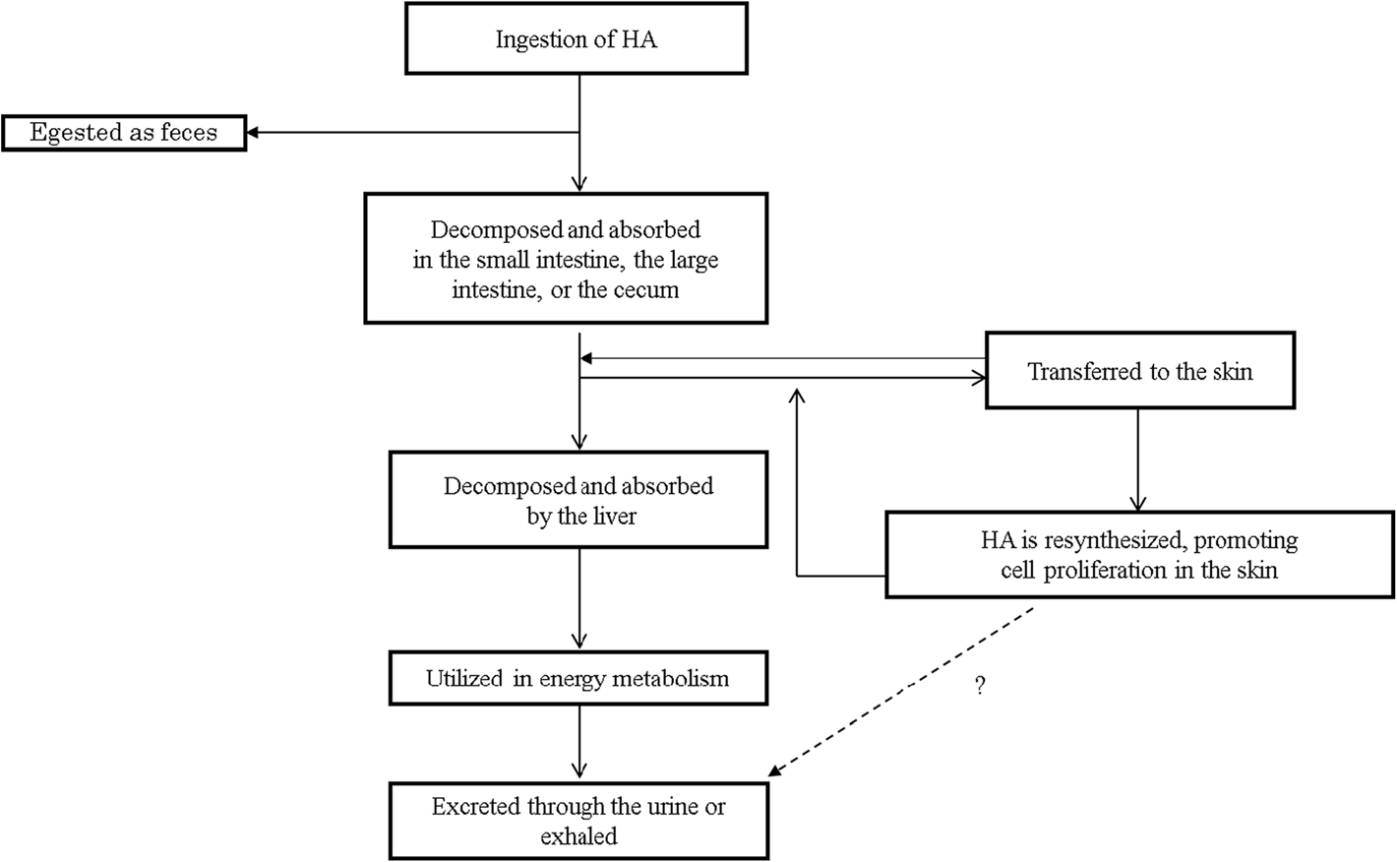
Metabolic pathway of ingested hyaluronan.

Polysaccharides can be degraded with digestive enzymes (e.g., starches) into monosaccharides or oligosaccharides, and these degraded fragments can then be absorbed. However, there are no digestive enzymes that degrade HA; thus, there is a possibility that HA is not degraded or absorbed in the body. However, several reports show evidence for the uptake and distribution of HA into the tissues (Figure 4). In the oral administration test of radioactively labeled, high MW HA (MW: 1 × 10^6^) to rats, approximately 90% of ingested HA was absorbed into the body and used by the body [43,44]. Subsequently, 80% of absorbed HA metabolites are excreted through urine and exhalation, and then decomposed and absorbed to utilize the source of energy. In contrast, the rest 10% HA metabolites remain in the body after utilization. Furthermore, approximately 10%, which was not absorbed into the body, were egested in the feces (in the orally ingested form). In addition, radioactively labeled, high- and low-MW HA (MW: 1 × 10^6^ and 1 × 10^5^, respectively) accumulated in skin tissue.

Ingested HA is believed to be absorbed by the intestinal route. The intestinal permeability of low MW HA was confirmed by cultured monolayers of human intestinal Caco-2 cells. Low MW HA was primarily permeated through the Caco-2 cell monolayer via the paracellular pathway and this permeation increased inversely with the molecular size of HA [45]. In addition, orally administered HA was decomposed into low molecules by intestinal bacteria in mice [46]. Bacteroides stercoris HJ-15, which potently degrades glycosaminoglycan [47], and bacteria such as Staphylococcus aureus [48] and Clostridium perfringens [49], which produce hyaluronidases, were found in the human intestine. Thus, in humans, consumed HA is also degraded by intestinal bacteria. Dietary fiber is a polysaccharide that is resistant to digestion and is similar to HA. It is partially degraded into short chain fatty acids such as propionic acid and butyric acid or into a monosaccharide by anaerobic fermentation of intestinal bacteria in the large intestine. Hence, ingested HA and dietary fiber can be degraded with the similar dissociation process. Therefore, high MW HA that is ingested should be degraded and absorbed by the body.

Balogh et al. showed high MW HA (MW: 1 × 106) was transferred into tissues without depolymerization when rats were orally administered radioactively labeled HA [44]. Further, Balogh et al. proposed that lymphatic uptake of orally administered HA initially occurs because its radioactivity in tissues preceded its appearance in the blood. However, consumed HA detected in the lymphatic fluid has not been analyzed yet.

These reports suggest that ingested HA should reach the skin by the blood and lymphatic transport systems. However, further studies need to elucidate the precise absorption mechanism of ingested HA.

### Mechanisms of action for the ingested hyaluronan

Consuming HA moisturizes the skin by causing the skin cells to transfer HA to the skin, despite its MW [50]. These absorption reports of digested HA reveal that partially depolymerized HA is absorbed by the gastrointestinal tract while intact HA is absorbed by the lymphatic system. Both the partially depolymerized and the fully intact HA were distributed to the skin. HA oligosaccharides (MW: 1–2 × 10^3^) increased HA production in human fibroblasts, probably by displacing endogenous HA from the receptors [51]. In addition, low MW HA used primers when high-molecular-weight HA was synthesized in the cortical cells of the vitreous body [52]. The amount of HA in the skin is one of the main factors that determines the skin moisture content [53]. The metabolites of ingested HA moisturizes the skin. High MW HA (MW: 1.1 × 10^6^) promoted cell proliferation in the manufacturing of human fibroblast populated collagen lattices [54]. This increase of the cell number suppresses the skin’s water loss by filling the gaps of the skin cells and increasing the amount of HA synthesis in the skin.

The aforementioned reports suggest that both low- and high-MW HA transfer to the skin and affect the fibroblast cells to promote HA synthesis and cell proliferation, which contribute to moisturizing the skin.

### Safety of ingested hyaluronan

HA is safe as a daily ingestible food. There are sufficient safety data for HA, no matter the origin or molecular weight (Table 2). Acute toxicity tests in animals reveal that oral HA ingestion results in a LD_50_ (lethal dose, 50%) of 800–2400 mg/kg, 200–1200 mg/kg, 900–1000 mg/kg body weight, or more, in mice, rats, and rabbits, respectively [36-38,55-57]. In a 28-day dietary HA (Hyabest®(S) LF-P: toxicity study, rats were orally administrated HA (0, 34, 235, 3536 mg/kg body weight/day). The results revealed no mortalities, no clinical observation of changes in body weight, no effects on food consumption or food efficiency, no changes in organ weight, gross findings, and no alterations in clinical pathology or histopathology [58]. Another study showed a no-observed-adverse-effect level (NOAEL) of more than 48 mg/kg per day in a 90-day dietary toxicity study of sodium hyaluronate [59]. Further, Ames tests revealed that no mutagenicity occurred in *Salmonella typhimurium* or *Escherichia coli* tester strains (≤1000 μg/plate HA) [60,61].

**Table 2:** Safety tests of hyaluronan

**Test procedure**	**Substance**	**Origin**	**Molecular weight**	**Subject**	**Result**	**Reference**
Daily oral administration test for 8 weeks	HA	Chicken comb	9 × 10^5^	Human	No abnormalities in hematology due to 240 mg/kg body weight/day for 12 weeks	Sato, T. *et al*. (2009) [63]
Daily oral administration test for 1 year	HA	Chicken comb	9 × 10^5^	Human	No abnormalities on clinical observations due to oral administration of 200 mg/kg body weight/day for 1 year	Tashiro, T. *et al*. (2012) [7]
Single-dose toxicity test	Sodium hyaluronate	Chicken comb	Not shown	Mouse	LD50 (mg/kg body weight) > 2400	Nagano, K. *et al*. (1984) [55]
					LD50 (mg/kg body weight) > 1200	Nagano, K. *et al*. (1984) [56]
	Sodium hyaluronate	Chicken comb	Not shown	Rat	LD50 (mg/kg body weight) > 800	Nagano, K. *et al*. (1984) [55]
					LD50 (mg/kg body weight) > 1200	Nagano, K. *et al*. (1984) [56]
		Microbial fermentation	16.8 × 10^5^		LD50 (mg/kg body weight) > 200	Morita, H. *et al*. (1991) [57]
	Sodium hyaluronate	Chicken comb	Not shown	Rabbit	LD50 (mg/kg body weight) > 1000	Nagano, K. *et al*. (1984) [55]
					LD50 (mg/kg body weight) > 900	Nagano, K. *et al*. (1984) [56]
Repeated-dose toxicity test	HA	Microbial fermentation	3 × 10^5^	rat	No abnormalities on clinical observations due to administration in doses equivalent to 0, 34, 235, 3536 mg/kg body weight/day.	Oe, M. *et al*. (2011) [58]
	Sodium hyaluronate	Chicken comb	Not shown		NOAEL 48 mg/kg body weight, or more	Ishihara, M. *et al*. (1996) [59]
Antigenicity test	Sodium hyaluronate	Microbial fermentation	18.8 × 10^5^	Bacteria (Ames test)	No mutagenicities to *S. typhimurium* (TA1535, TA1537, TA98, or TA100) or *E.coli* (WP 2 urA)	Sugiyama, C. *et al*. (1991) [60]
		Not shown	21.2 × 10^5^		No mutagenicities to *S. typhimurium* (TA98, TA100, TA1535, or TA1537) or *E.coli* (2 urA)	Onishi, M. *et al*. (1992) [61]
		Chicken comb	20.0–21.2 × 10^5^	Mouse, rat, rabbit	No antigenicity on PCA reaction in mice or guinea pigs	Takemoto, M. *et al*. (1992) [62]
					No antigenicity on active systemic anaphylactic reaction in guinea pigs	

Furthermore, sodium hyaluronate was found to be negative in antigenicity tests in both mouse–rat and guinea pig–guinea pig systems of passive cutaneous anaphylaxis (PCA) reaction [62]. The active systemic anaphylaxis reaction induced by HA was found to be negative in the guinea pig used in the guinea pig-guinea pig system of PCA reaction [62].

The safety of ingested HA was validated in human clinical trials (unpublished data). In a randomized, double-blind, placebo-controlled study, human subjects (average age ± S.E.; 30.2 ± 9.7) that had rough and chronically dry skin received low or high doses of HA (Hyaluronsan HA-F, 120 mg/day, n = 17; 360 mg/day, n = 17, respectively) or a placebo, crystalline cellulose (n = 18) for 4 weeks. The effects of ingested HA on the human body were examined by the blood test. Significant changes were observed in parts of blood components compared before ingestion in all groups; however, these changes were within normal limits and were not considered a medical abnormality. Further, hematological abnormalities did not occur in human subjects who received 240 mg/day and 200 mg/day of HA (Hyabest®(J): MW 9 × 10^5^, Kewpie Co., Tokyo) for 12 weeks or 12 months, respectively [7,63].

These clinical trials show that HA is a safe dietary supplement that does not harm the body.

## Conclusion

The reduction of HA in the skin by intrinsic and extrinsic factors such as aging and ultraviolet radiation, smoking and air pollutants induce dryness in the skin. However, daily HA supplements can moisturize the skin because the metabolites of HA increases the skin moisture content by having an effect on the skin cells. Thus, consuming HA affects skin cell and improves dry skin physiologically. This review shows that consuming HA moisturizes the skin and employing HA as a dietary supplement makes the skin healthy. We believe that countries worldwide will benefit from this review and consume HA to alleviate dry skin.

## Abbreviations

HA: Hyaluronan; HAS: Hyaluronan synthases; NOAEL: No observed adverse effect level; MW: Molecular weight; LD_50_: Lethal dose 50%; PCA: Passive cutaneous anaphylaxis.

## Competing interests

The authors declare that they have no competing interests.

## Authors’ contributions

All authors managed the literature searches, formulated the hypothesis and contributed to the discussion and conclusions. CK mainly wrote the manuscript. All authors read and approved the final manuscript.
